# Two Cases Exemplifying the Powers and Uses of the Borat of Mercury

**Published:** 1802-08-01

**Authors:** 


### *Two Cases exemplifying the Powers and Uses of the Borat of Mercury.*

WHEN we pour nitrat of mercury on a ſolution of the borat of ſoda, or borax, we obtain a yellow precipitate, which is the Borat of Mercury. This compound has not been much attended to, but it ſeems evidently to poſſeſs active powers on the human body, whether given internally or applied externally. When given internally, it has all the powers of ſome of the other mercurial preparations, as the hydrargyrus phoſphoratus, cinereus, &c. It produces ſalivation, and alſo acts as a gentle laxative. It may be given internally in the form of pills, beginning with a doſe not exceeding one grain.

When applied externally, it ought to be mixed with axunge, and rubbed in, in the ſame way as other mercurial ointments; it ſeems alſo to form a good dreſſing for venereal ſores.

The two following caſes are the only ones in which I have had an opportunity to employ it, and in both it has exhibited the ſame effects.

T—— R——, aged 18 years, was, on the 15th of June laſt, attacked with lues venerea, which appeared in the form of chancres on the glans penis. He applied to me on the 18th. I ordered him to dreſs them with an ointment, compoſed of hogs lard and the borat of the mercury, and I gave him ſome pills, containing each half a grain of the powder; of theſe he was to take two at bed time. I did not ſee him again for about a week, when the chancres had put on a healthy appearance, and had contracted a little in ſize; but a ſmall tumour had appeared in the left groin, attended with ſome pain. He had begun to ſalivate. I ordered him to increaſe the doſe of the pills; to rub in the ointment with which he dreſſed the chancres, and to apply 6 or 8 leeches to the tumour. It was about a fortnight after, when I ſaw him again, when the chancres were leſs, as alſo the tumour in the groin. He told me he had applied the leeches again, and had likewiſe been uſing the common blue ointment. He was completely cured in about five weeks after, during which time the leeches had been applied to the tumour in the groin four different times; and he had uſed about three ounces of the common mercurial ointment. In this caſe I ſhall not ſay the patient recovered entirely by the uſe of the borat, but I have no doubt but it aſſiſted conſiderably.

The other Caſe is the following:

Mr. R—— C——, aged 24, came to me on the 10th of December laſt, with two ulcers in his throat, and alſo a very ill ill conditioned one on the elbow joint. They had all the appearance of venereal ulcers. He had been affected with lues about half a year before, when he had a bubo in each groin, one of which fuppurated. He had been cured by the ufe of mercury. He faid, that the ulcers had appeared in his throat about a fortnight ago, after a common catarrh, and that the ulcer had broke out on his elbow, after a fall on that part. The fore was dreffed with the ointment prepared as above. He took alfo fome of the pills; rubbed in on the thigh a fmall quantity of common mercurial ointment, and ufed a gargle of oak bark. He recovered in about fix weeks. Thefe are all the obfervations I have as yet to make; if, afterwards, they fhall be confirmed by experience, I fhall be exceedingly happy to hear of it.

*Edinburgh, Feb.* 9, 1802. A. R.

P. S. I have to inform you, that Dr. Monro has this winter opened a private Diffecting Room for the ftudents, in which a great number can be accommodated. In addition to it, there are fome others, particularly that of Dr. Barclay's.

